# A New FPGA Architecture of FAST and BRIEF Algorithm for On-Board Corner Detection and Matching

**DOI:** 10.3390/s18041014

**Published:** 2018-03-28

**Authors:** Jingjin Huang, Guoqing Zhou, Xiang Zhou, Rongting Zhang

**Affiliations:** 1 School of Precision Instrument & Opto-Electronics Engineering, Tianjin University, Tianjin 300072, China; jingjin_huang@tju.edu.cn (J.H.); zrt65@tju.edu.cn (R.Z.); 2 Guangxi Key Laboratory for Spatial Information and Geomatics, Guilin University of Technology, Guilin 541004, China; 3 School of Microelectronics, Tianjin University, Tianjin 300072, China; zqx0711@tju.edu.cn

**Keywords:** FPGA architecture, FAST, BRIEF, corner feature, detection & matching

## Abstract

Although some researchers have proposed the Field Programmable Gate Array (FPGA) architectures of Feature From Accelerated Segment Test (FAST) and Binary Robust Independent Elementary Features (BRIEF) algorithm, there is no consideration of image data storage in these traditional architectures that will result in no image data that can be reused by the follow-up algorithms. This paper proposes a new FPGA architecture that considers the reuse of sub-image data. In the proposed architecture, a remainder-based method is firstly designed for reading the sub-image, a FAST detector and a BRIEF descriptor are combined for corner detection and matching. Six pairs of satellite images with different textures, which are located in the Mentougou district, Beijing, China, are used to evaluate the performance of the proposed architecture. The Modelsim simulation results found that: (i) the proposed architecture is effective for sub-image reading from DDR3 at a minimum cost; (ii) the FPGA implementation is corrected and efficient for corner detection and matching, such as the average value of matching rate of natural areas and artificial areas are approximately 67% and 83%, respectively, which are close to PC’s and the processing speed by FPGA is approximately 31 and 2.5 times faster than those by PC processing and by GPU processing, respectively.

## 1. Introduction

The detection and matching of feature points are one of the most key steps in satellite image applications, such as image registration, image mosaic, change detection, geometrical calibration, 3D reconstruction and object tracking & recognition [1]. Therefore, the performance of the detection and matching algorithm directly influences its applications. Various impressive algorithms [2,3,4,5,6,7,8] have been proposed in recent decades, such as Scale Invariant Feature Transform (SIFT) [9], Speeded Up Robust Features (SURF) [10], Oriented FAST and Rotated BRIEF (ORB) and KAZE features [11]. Most of these algorithms perform well on the PC under the indoor implementation. With the increasing requirement of real-time processing of satellite imagery in, such as, natural disasters detection and monitoring, public security and military operation [12,13], these algorithms cannot meet the requirement of high performance of real-time on-board processing. Therefore, it is attracting scientists’ interests on the real-time detection and matching of feature points in satellite imagery.

Currently, satellites operate under stringent constraints on volume, power, memory and computational burden. A new image processing platform, which has a low volume, low power and high throughput, is required. In this instance, a Field Programmable Gate Array (FPGA), which can offer a highly flexible design and a scalable circuit, is selected as the hardware platform. The pipeline structure and the fine-grained parallelism of FGPA have strengths in processing on the pixel level. The size and speed of FPGA are comparable to ASIC. Meanwhile, the design of FPGA is more flexible and its design cycle is shorter [14]. Therefore, an FPGA implementation for detection and matching is proposed. The implementation means that the corresponding algorithms are transformed into hardware circuits, which run in an FPGA chip of an embedded system. Very-High-Speed Integrated Circuit Hardware Description Language (VHDL) or Verilog HDL was adopted as the hardware description language in the transformation process.

A few researchers have investigated the FPGA implementation of features detection and/or matching for real-time applications, such as: (1) In the feature detection, many recognized algorithms of multi-scale feature point detectors were mapped into FPGA, such as SIFT detector [15], the optimized SIFT detector [16], SURF detector [17,18], OpenSURF detector [19,20]. Corner and/or dot detectors are also mapped into FPGA, such as SUSAN [14], the optimized SUSAN [21], Harris [22], FAST [23,24,25] and the machine-learned FAST [26]. An FPGA implementation of Sobel edge detector for line feature was proposed in [27] and a polygon feature was first mapped into FPGA by [28]; (2) In the feature matching, many appropriate descriptors have been mapped into FPGA, such as the SURF descriptor [17,29,30], the modified SURF descriptor [31], the BRIEF descriptor [15,18,24,32], the rotated BRIEF [33] and the SIFT descriptor [34]. In an FPGA implementation of feature detection, the multi-scale feature point, line feature and polygon feature usually consume many FPGA resources, resulting in a poor real-time performance, due to the complicated algorithms and the floating-point arithmetic. For instance, reference [34] implemented the SIFT descriptor on FPGA but it is high computational cost, because of its orientation calculation and 128 dimensional descriptors. The SIFT descriptor is usual implemented on DSP [35] or NIOS II software core [16,36]. Although the SURF descriptor can achieve a similar performance when compared to the SIFT descriptor and can be mapped into FPGA by fixed-point arithmetic, the consumption of FPGA resources is relatively large [19].

The corner is one of the most distinguishable feature points, which are repeatable and robust and have an accurate and stable projection between 3D and 2D spaces [25]. Meanwhile, the satellite image can be located in various objects, such as in buildings, in coastlines, in mountain ridges and in ridges and so forth. Based on the analysis mentioned, this paper presents a complete solution for corner detection and matching on-board satellite. The proposed solution is based on the combination of the FAST [6] and BRIEF algorithm [8]. The combined algorithm implemented into FPGA is capable of continuous processing of an incoming image sequences. There are a few investigations on an FPGA implementation of FAST and BRIEF algorithm. For example, Heo et al. (2013) proposed an FPGA implementation of the FAST and BRIEF algorithm for object recognition. To save hardware resources, the FAST detector, the corner score module and BRIEF module were executed in order and the speed was approximately 55 frames per second (fps) [24]. Fularz et al. (2015) also implemented the FAST and BRIEF algorithm on FPGA. To increase the fps, the authors achieved the corner detection module and BRIEF generation module in parallel, without the DDR3 write/read module. The speed of FPGA architecture can reach 325 fps [25]. In reference [24], the FPGA architecture is designed as a low fps for saving hardware resources. In reference [25], the image data were not first stored in memory but directly sent to a FAST detection module and a BRIEF generation module at the same time. Such an operation resulted in an inability to reuse the image data by follow-up algorithms, such as sub-pixel precision location. To ensure a high frame rate in data processing and reuse of image data, this paper proposes a new FPGA architecture for on-board implementation of the FAST and BRIEF algorithm. In the architecture, a remainder-based method is firstly proposed to read a sub-image at a minimum cost. The six pairs of images with different textures are used to evaluate the performance of the FPGA implementation.

The paper is organized as follows: Section 2 gives a brief overview of the corner detection and matching algorithms. The FPGA architecture and its implementations are presented in Section 3. Section 4 presents the results and the performance of the experiment. Section 5 is a discussion of the results. Finally, Section 6 concludes this work and makes recommendations for future research.

## 2. Overview of the FAST and BRIEF Algorithm

A combination of the FAST detector and the BRIEF descriptor is presented in this section. The fundamental ideas of this algorithm are as follows: (i) the corners which are repeatable and robust are detected at pixel-level precision; (ii) a sub-image centered on the detected corner is extracted from Double Data Rate (DDR) 3 SDRAM and then, a BRIEF descriptor is generated; (iii) the corresponding point pair is identified by finding the minimum value of candidate Hamming distances. This combination algorithm can be broken down into three steps, which are described individually in the following sections.

### 2.1. FAST Detector

**Figure 1:**
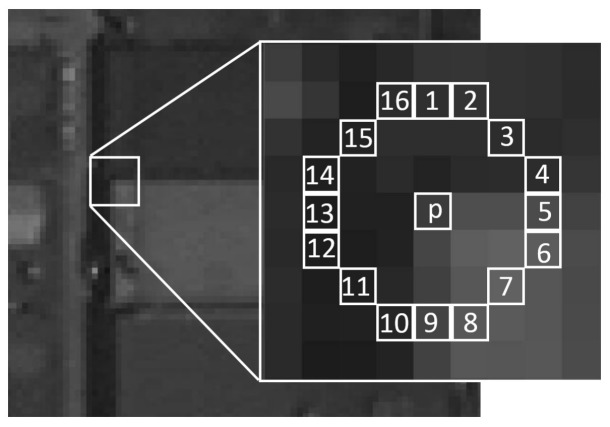
FAST detector.

The FAST detector first proposed by Rosten [2] is widely used in corner detection for computer vision because of a rapid operation and low computations compared to other corner detectors. The segment test criterion operates by analyzing a circle of sixteen pixels around the candidate corner *p*—as illustrated in Figure 1—the original detector classifies *p* as a corner if there exists a set of *n* contiguous pixels in the circle, which are brighter than the intensity of the candidate corner *Ip* plus a threshold *t*, or are darker than *Ip* minus the *t*. The *n* is chosen as 12 because it allows a high-speed test that can be used to exclude a very large number of non-corners.

The formula of the FAST detector is presented as follows:

(1) $$S_{p\to x}=\{\begin{array}{ccc} d, & I_{p\to x}\leq I_{p}-t & \left(\mathrm{darker}\right)\\ s, & I_{p}-t<I_{p\to x}<I_{p}+t & \left(\mathrm{similar}\right)\\ b, & I_{p}+t\leq I_{p\to x} & \left(\mathrm{brighter}\right) \end{array}$$

where *I_p_* is the intensity of *p*, *I_p_*_→*x*_ is the intensity of the sixteen pixels around the corner and *t* is a threshold. If *S_p_*_→*x*_ is equal to *d*, the pixel belongs to the darker group; if *S_p_*_→*x*_ is equal to *s*, the pixel belongs to the similar group; if *S_p_*_→*x*_ is equal to *b*, the pixel belongs to the brighter group. If there exist 12 continuous pixels that belong to the darker or brighter group, *p* is regarded as a corner.

When all image pixels are tested using the above process, the corners are determined. The corners will converge in some areas. To find the most robust corners, a non-maximal suppression method based on a score function is adopted. The score values of each detected corner are calculated and then, the corners with the lower score values are removed and the corners with higher score values are kept using the non-maximal suppression method. There are several intuitive definitions for the score value:

(1) The maximum value of *n* for which *p* is still a corner;
(2) The minimum value of *t* for which *p* is still a corner;
(3) The sum of the absolute difference between the pixels in the contiguous arc and the center pixel.

Definitions (1) and (2) are highly quantified measures and many pixels share these same values. For the speed of computation, a slightly modified version of (3) is used. The score value is calculated as follows:

(2) $$score=max\left(\sum_{x\in S_{bright}}\left|I_{p\to x}-I_{p}\right|-t,\sum_{x\in S_{dark}}\left|I_{p}-I_{p\to x}\right|-t\right)$$

where *I_p_* is the intensity of *p*, *I_p_*_→*x*_ is the intensity of the sixteen pixels around the corner and *t* is a threshold.

### 2.2. BRIEF Descriptor

The BRIEF descriptor first proposed by M. Calonder [8] is adopted to describe the detected corner. The form of the BRIEF descriptor consists of “1” and “0”, and the length of the BRIEF descriptor is generally defined as 128 bits, 256 bits and 512 bits, which are efficiently implemented by FPGA with a low consumption. The following formula clearly shows the definition of the BRIEF descriptor:

(3) $$\lambda \left(p;r_{1},c_{1},r_{2},c_{2}\right)=\{\begin{array}{c} 1:I\left(r_{1},c_{1}\right)<I\left(r_{2},c_{2}\right)\\ 0:I\left(r_{1},c_{1}\right)\geq I\left(r_{2},c_{2}\right) \end{array}$$

where *I*(*r*_1_, *c*_1_) and *I*(*r*_2_, *c*_2_) are the intensity of the pixels at (*r*_1_, *c*_1_) and (*r*_2_, *c*_2_). If *I*(*r*_1_, *c*_1_) is less than *I*(*r*_2_, *c*_2_), then *λ* = 1; otherwise, *λ* = 0. The length of *λ* is designated as 256 bits in this paper.

**Figure 2:**
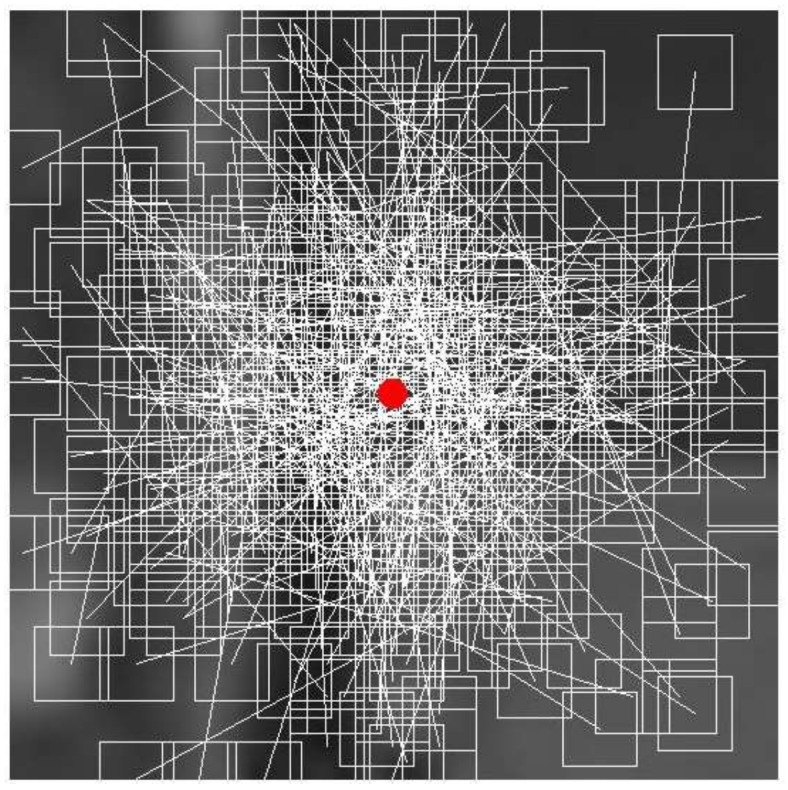
256 patch-pairs of BRIEF descriptor.

In the description of one point, a sub-image with a size of 35 columns × 35 rows (here, the definition in the following situation is the same) is used. Because the BRIEF descriptor is sensitive to noise, the intensity value of the patch-pair is calculated using a smoothing filter with a 5 × 5 sub-window centered on (*r_i_*, *c_i_*), (*i* = 1, 2, … and 512) (see Figure 2). To reduce the impact of the image boundary, the intensity values of the image boundary are removed from the computation and thus, the actual size of the sub-image is reduced to 31 × 31. Next, {(*r*_1_, *c*_1_), (*r*_2_, *c*_2_)} is defined as a patch-pair instead of a point-pair and there is a total of 256 patch-pairs in the sub-image. The locations (*r_i_*, *c_i_*) of the 256 point pairs are determined by the Gaussian distribution. (*r_i_*, *c_i_*)~i.i.d. Gaussian (0, *S*^2^/25):(*r_i_*, *c_i_*) are determined from an isotropic Gaussian distribution; *S* is the size of a patch. Details for how to determine the locations of the 256 patch-pairs can be found in [8].

### 2.3. Corner Matching

When finishing the generation of descriptors of the detected corners, we need to determine the correctly matching corners in two images by using Hamming distance. A Hamming distance is the number of different characters in the corresponding position of two character strings. If the Hamming distance equal to “*n*” (*n* ≥ 0), it means that there are “*n*” different characters. In general, if “*n*” is less than a threshold “*t*”, we will identify the corresponding two character strings are same.

In corner matching phase, the character string is the BRIEF descriptor (a binary vector). The number of different characters is calculated by an XOR operation [8]. Table 1 clearly presents the matching process of two different images by Hamming distance. For instance, three Hamming distances between one descriptor (ID = 1) from the second image and three descriptors (ID = 1, 2, 3) from the first image are calculated by XOR operation. The minimum value of the calculated distances means the corresponding two descriptors are the most similar. If the minimum value is less than a given threshold that indicate the corresponding corners are matched.

## 3. Proposed FPGA Architecture

### 3.1. The Whole Architecture

**Figure 3:**
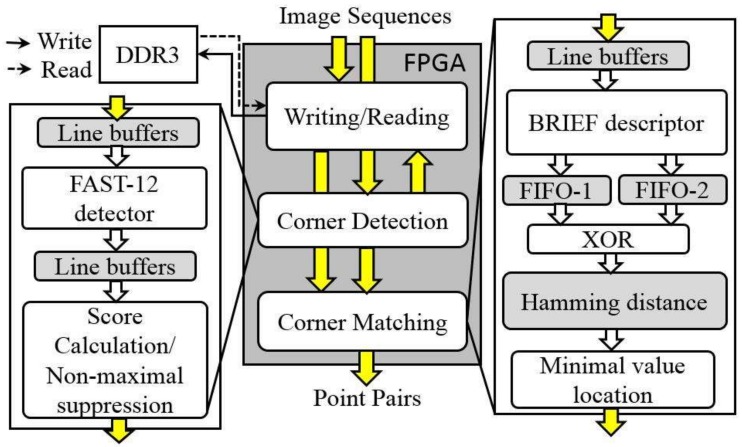
FPGA architecture of corner detection and matching.

To achieve on-board detection and matching of satellite images, a new architecture mapped in a single FPGA chip (see Figure 3) is proposed. The architecture consists of three main modules: Writing/Reading, Corner Detection and Corner Matching. Each module is briefly described as follows:

(1) The Writing/Reading module controls the writing/reading of image data and generates the corresponding writing/reading addresses. The image data are stored into a DDR3, which is 512 Mb of external memory.
(2) In the Corner Detection module, the image data input from the image sequences are sent into line buffers and then, the candidate corners are first located using the FAST algorithm, which is implemented by using 16 comparators. The most robust corners are determined by a non-maximal suppression sub-module.
(3) In the Corner Matching module, when the most robust corners are output, the locations of the corners are sent to the Writing/Reading module to read the corresponding sub-images centered on the corners. The sub-images are used to generate the BRIEF descriptors. Each BRIEF descriptor consists of a binary vector. The BRIEF descriptors in the first image are sent into First In First Out (FIFO)-1 and the BRIEF descriptors in the second image are sent into FIFO-2. The Hamming distances between the BRIEF descriptors stored in FIFO-1 and FIFO-2 are calculated. A point pair with the minimal Hamming distance is output as the final result.

### 3.2. Writing/Reading Module

**Figure 4:**
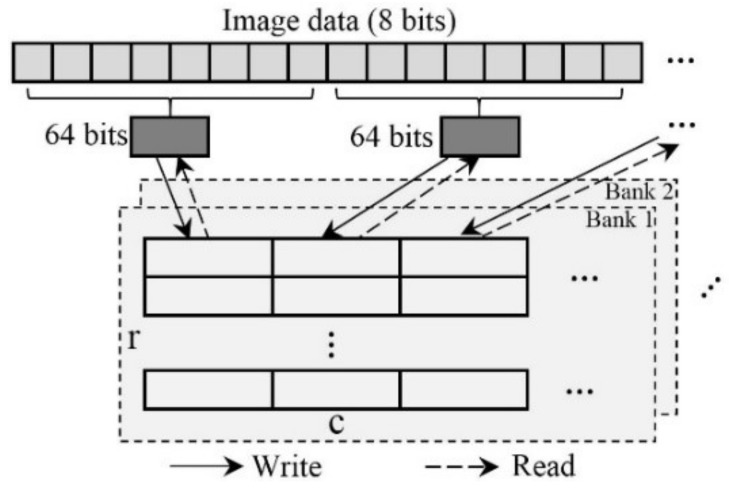
Writing/reading module.

To write the image data into DDR3 and read them out successfully, a Writing/Reading module is essential when it operates a DDR3 IP. Here, the six control signals (*app_cmd*, *app_addr*, *app_en*, *app_wdf_data*, *app_wdf_wren*, *app_wdf_end*) need to be re-designed on the basis of relative modules [37]. In a parameters setup state, if the burst length of the DDR3 is 8 and the data width is defined as 8 bits, the writing/reading data width should be 64 bits. The processes of writing/reading are presented in Figure 4. As seen in Figure 4, the data with 64 bits, which combine 8 image data with 8 bits, are written into a cell bank based on the writing address. According to the reading address, the data with 64 bits are read out from a cell bank and then separated into 8 image data with 8 bits. The writing/reading addresses determine which cell is writing and reading.

### 3.3. Corner Detection Module

**Figure 5:**
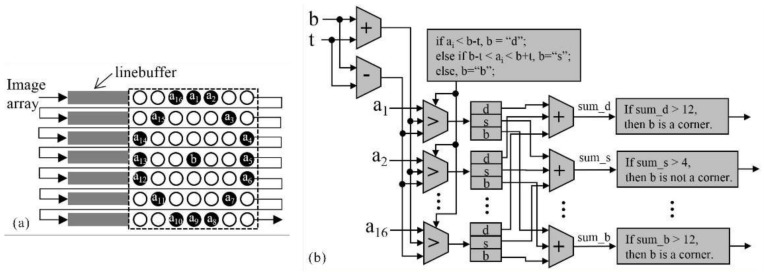
FAST detector module, (**a**) line buffers; (**b**) FAST-12.

The image data input from the image sequences are sent into line buffers (namely, RAM-based shift registers in IP core) with a 512 bits depth. The following FPGA architecture presents the pipeline-based operation of FAST-12 detector. As seen from Figure 5a, “*b*” is a candidate corner and “*a_i_*” is comparative point. When there exist 12 contiguous “*a_i_*” that are greater than “*b+t*” or less than “*b−t*,” “*b*” is defined as a corner. Otherwise, “*b*” is not a corner.

To find the 12 continuous “*a_i_*” mentioned above, the pipeline-based processing is shown in Figure 5b. As seen in Figure 5b, “*b+t*” and “*b−t*” are first calculated and then, the calculated results and “*a_i_*” are sent into comparators. Here, 16 channel comparators are parallel processing in the same system clock. Details of the comparison are listed as follows:

If “*a_i_*” is less than “*b−t*,” then “*a_i_*” belongs to “*d*” (darker);

If “*a_i_*” is greater than “*b−t*” and less than “*b+t*,” then “*a_i_*” belongs to “*s*” (similar);

Otherwise, “*a_i_*” belongs to “*b*” (brighter).

After finishing the comparison mentioned above, the numbers of “*d*,” “*s*” and “*b*,” are counted. When there exists over 12 continuous “*a_i_*” that belong to “*d*” or “*b*,” the candidate point is output as a corner.

**Figure 6:**
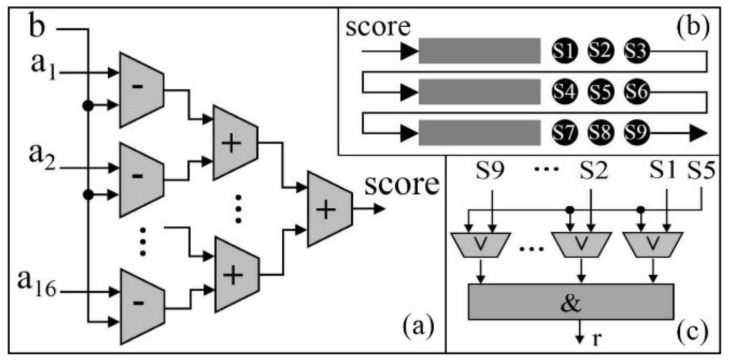
Robust corner selection module, (**a**) corner score; (**b**) line buffers; (**c**) non-maximum suppression.

Using the above processes, the corners are determined with surprising speed. However, the process identifies corners that are too clustered in some areas. To identify the corners with more robust characteristics, the score of the point (“*b*”) is calculated. The pipeline-based implementation of the score formula (Equation (2)) is presented in Figure 6a. As presented in Figure 6a, an “adder tree” architecture is used. For example, the differences between “*b*” and “*a_i_*” are calculated using 16 subtractors in the first level and the sums of the differences are processed by 8 adders in the second level, 4 adders in the third level, 2 adders in the fourth level and 1 adder in last level. Once the calculation is completed, the score values are sent into the line buffers with a depth of 512 bits (see Figure 6b) and then, a non-maximum suppression is identified with a size of 3 × 3. In non-maximum suppression processing (Figure 6c), “S5” is compared with the other 8 score values by 8 comparators and the comparison values are calculated using logical conjunction. If the result “*r*” is equal to 1, “S5” is greater than the other 8 score values and is kept as a more robust corner. Otherwise, “S5” is removed from the corner sets. Additionally, the score value of non-corner is defined as 0.

### 3.4. Corner Matching Module

**Figure 7:**
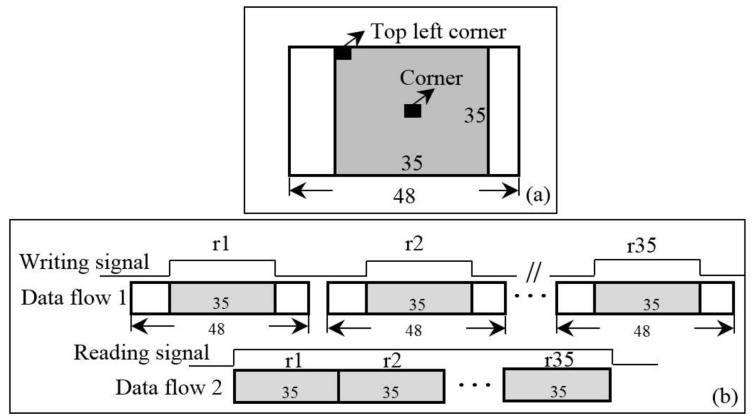
Image cutting module, (**a**) the location of top left corner determination; (**b**) a smaller sub-image clipping.

**Figure 8:**
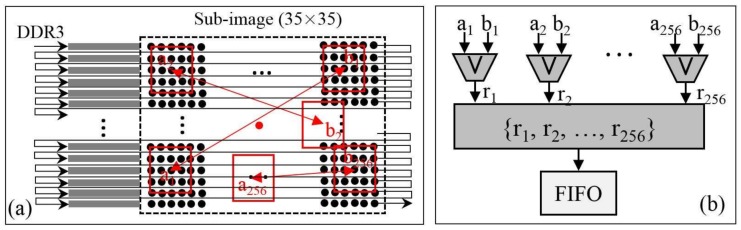
BRIEF descriptor generation module, (**a**) line buffers; (**b**) 256 binary vector.

When the columns and rows of corners are located, the sub-images centered on the corners are used for the BRIEF descriptors generation. To read the sub-images from the bank of DDR3, the corresponding reading addresses are generated according to the rows and columns of corners. Here, details of a BRIEF descriptor generation are listed as follows:

(1) The burst length of DDR3 is 8 and the size of the sub-image is 35 × 35. Hence, the smallest size of a sub-image needed to be read is 48 × 35. To cut out the sub-image with 35 × 35 from the sub-image with 48 × 35, a remainder-based method first proposed in this paper is adopted. Details of the remainder-based method are presented in Figure 7:
  - First, locate the column of the top left corner of the smaller sub-image (Figure 7a), the value of the remainder is calculated by dividing the column by 8. In an FPGA implementation, the divider can be replaced with the right shift operation;
  - According to the calculated remainder, the writing signal is active-high between the (remainder)th and (remainder+35)th data at each row when the sub-image (48 × 35) is written into FIFO. Then, the smaller sub-image (35 × 35) is output when the read signal is active-high (Figure 7b).
(2) In the BRIEF descriptor module, the sub-image of 35 × 35 reading from FIFO are sent into line buffers with a depth of 35 bits (see Figure 8a). A box filter with a size of 5 × 5 is performed on the sub-image. Then, the 256 patch-pairs are selected on the basis of the filtered sub-image. The FPGA implementation of Equation (3) is presented in Figure 8b. As presented in Figure 8b, the 256 patch-pairs are compared to generate a binary vector. The 256 comparators are processed in parallel and a combination operation is used to combine a BRIEF descriptor with 256 bits. The BRIEF descriptor is stored into the FIFO unit waiting for matching processing. Because of the fixed-point arithmetic of Equation (3), the FPGA is simple to implement in parallel, which makes the BRIEF algorithm attractive for use in real-time image processing.

**Figure 9:**
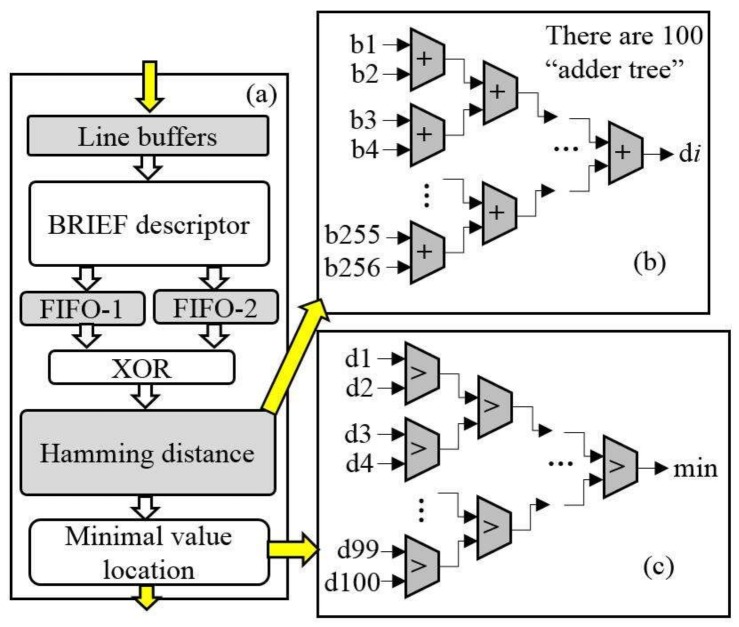
Matching module, (**a**) matching processing; (**b**) adder tree; (**c**) finding minimum value.

The FPGA implementation of corner matching is presented in Figure 9. As seen in Figure 9a, the BRIEF descriptors in the first image and the second image are stored into FIFO-1 and FIFO-2, respectively. To reduce running time and save hardware resources, the maximum number of BRIEF descriptors is defined as 100. The results of the XOR operation between the first BRIEF descriptor in second image and the 100 BRIEF descriptors in first image are sent to 100 Hamming distance modules in parallel. One Hamming distance module is presented in Figure 9b. As shown in Figure 9b, one value is calculated using the “+” operation with each bit of the result of the XOR operation. Because there are 100 BRIEF descriptors in the first image, 100 Hamming distances are output at the same time. The 100 Hamming distances are sent to the minimal value location module (see Figure 9c) to find the minimal value location module. In this module, each “>” operation is used to find the smaller of two input data. The first BRIEF descriptor in the second image and the one in the first image with the smallest value are matched and output as a point pair. To match the latter BRIEF descriptor in the second image and one in the first image, the processes are the same as in the first BRIEF descriptor in the second image.

## 4. Experiment and Analysis

### 4.1. Hardware Platform and Test Field

Advances in programmable logic devices have resulted in the development of FPGA, which allows a large number of programmable logic elements to be placed on a single chip. An FPGA chip is an array of logic blocks placed in an infrastructure of interconnections, which can be programmed at three distinct levels: the function of the logic blocks, the interconnections among blocks and the inputs and outputs. An FPGA is programmable at the hardware level, thus combining the advantages of both general-purpose processors and specialized circuits [38]. In this paper, the hardware platform contains a Xilinx XC72K325T FPGA that is produced by Xilinx Company. The selected FPGA has 326,080 Logic Cells, 4000 kb Block RAM and 840 DSP Slices [39]. The resources of this board are sufficient to implement the proposed design. In addition, the designed tool is a Vivado 2014.2, the simulation tool is a Modelsim-SE 10.4 and the hardware design language is Verilog HDL.

**Figure 10:**
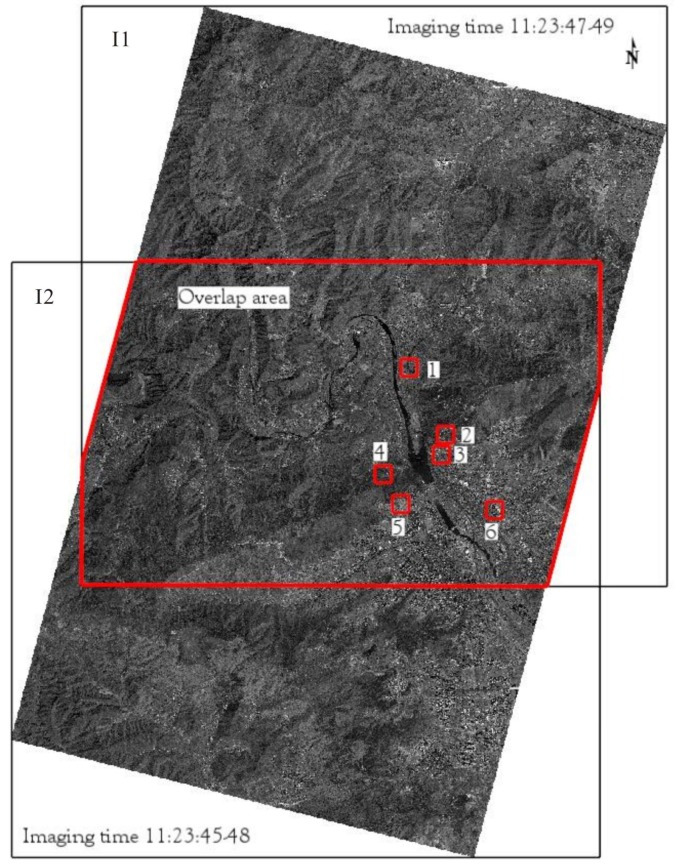
Test field in Mentougou district, Beijing, China. (1) expressway; (2) rural road; (3) bungalow; (4) tree; (5) bare soil; (6) high-rise buildings.

Six image pairs produced by GJ-1-01/02 on 6 May 2017 are used to evaluate the performance of the FPGA implementation of the FAST and BRIEF algorithm. In Figure 10, the six image pairs with a spatial resolution of 0.5 m are located in the Mentougou District, Beijing, China. The six image pairs represent different ground objects. The relationship between two images can be descripted by the Homography matrix. The locations of feature points in the first image can be used to calculate the corresponding locations in the second image by the homography matrix. For instance, once the rows and columns of the corners in first image are detected, the corresponding rows and columns in second image are determined by the calculated homography matrix. The rows and columns of corners in two images can used as the given value. Hence, to quantify the accuracy of the matching, the homography matrices of the six image pairs are calculated in advance by OpenCV (2.4.9 version) [40] on a PC. The calculated results are listed as follows.

(4) $$H_{1}=\left[\begin{array}{ccc} 1.0063671269362660 & -0.0025084112191981 & -5.3603942530664659\\ 0.0024154619306485 & 1.0006896930121771 & 1.0701971332427289\\ 2.3547190871544529e-05 & -1.4068295335751301e-05 & 1 \end{array}\right]$$

(5) $$H_{2}=\left[\begin{array}{ccc} 0.9943105109179883 & 0.0053035459466818 & -7.9850738811950164\\ -0.0049146471348655 & 1.0009732657999768 & 19.720810703664444\\ -3.3584978590059181e-05 & 3.0843312736350301e-05 & 1 \end{array}\right]$$

(6) $$H_{3}=\left[\begin{array}{ccc} 1.0009392303075071 & -0.0001790798237475 & 3.0604214830362744\\ 0.0006233318520506 & 0.9995032891288868 & 0.0381502563166876\\ 2.7656023979311547e-06 & -2.6050872046892108e-06 & 1 \end{array}\right]$$

(7) $$H_{4}=\left[\begin{array}{ccc} 1.0007150298250256 & 0.0006961964858254 & 3.1250680387908756\\ -0.0012117721797761 & 0.9996919849732843 & 2.4276670954674198\\ -1.1455611046402165e-06 & 2.4465459033581869e-06 & 1 \end{array}\right]$$

(8) $$H_{5}=\left[\begin{array}{ccc} 0.9871625029439816 & -0.0036529604851436 & 1.4444554238703597\\ -0.0077799496384017 & 0.9899688901024329 & 5.6760993259601014\\ -4.5234691072258501e-05 & -5.9335789302099768e-06 & 1 \end{array}\right]$$

(9) $$H_{6}=\left[\begin{array}{ccc} 1.0016636661740776 & 0.0009195592101046 & -0.9562208024016972\\ 0.0006934477656932 & 1.0009886508407932 & -1.9128674018458289\\ 4.0160183632567340e-06 & 2.3466827738924636e-06 & 1 \end{array}\right]$$

### 4.2. Experiment Results

**Figure 11:**
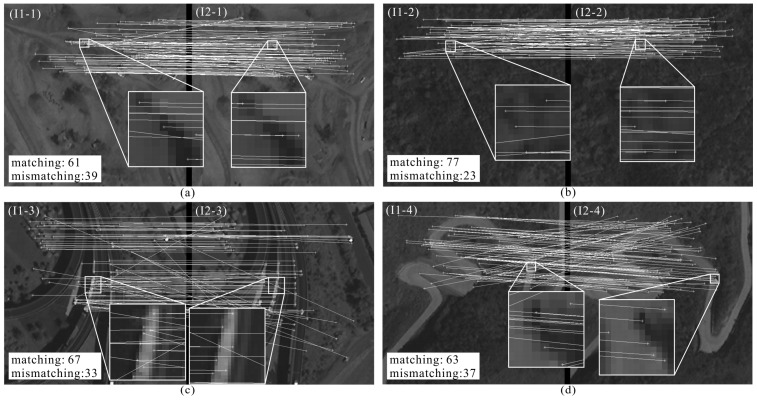

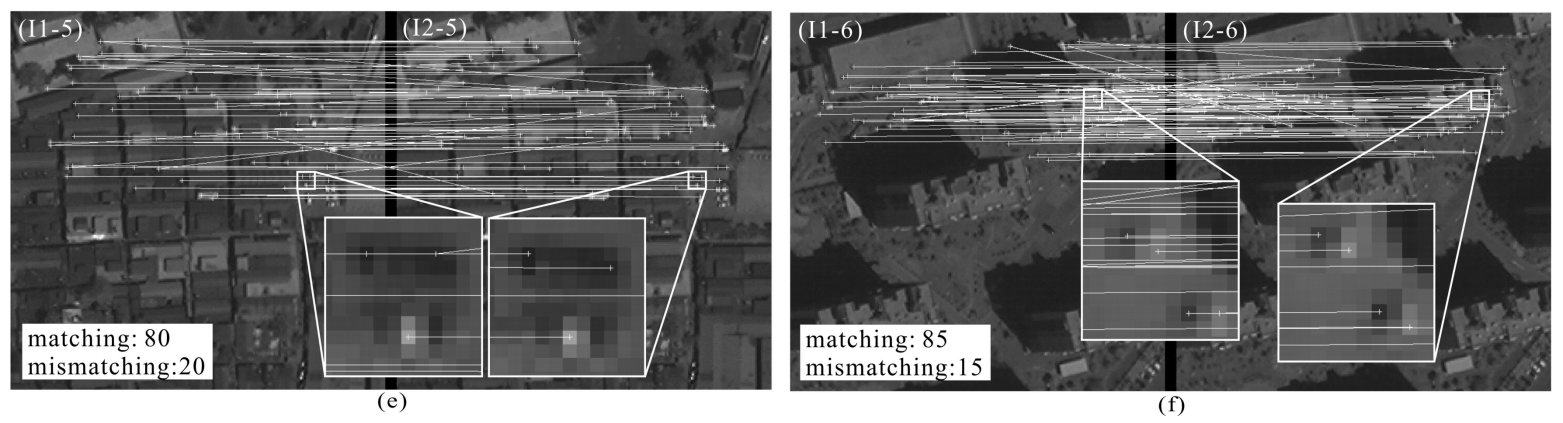
Matching results of six image pairs, (**a**) bare soil; (**b**) trees; (**c**) expressways; (**d**) rural roads; (**e**) bungalows; (**f**) high-rise buildings.

The results of the experiment using MATLAB software (R2014a version) are displayed in Figure 11. As seen in Figure 11a,b when the image pairs are covered with bare soil and trees, the numbers of correctly point pairs are 61 and 77; Figure 11c,d when the image pairs are covered with traffic lines, such as expressways and rural roads, the numbers of correctly point pairs are 67 and 63; Figure 11e,f when the image pairs are covered with buildings, such as bungalows and high-rise buildings, the numbers of correctly point pairs rise to 80 and 85. The results indicate that the matching rate is impacted by the textures, especially when the image is covered with buildings, a high matching rate is achieved. A further analysis of the matching performance is depicted in next section.

### 4.3. Accuracy Analysis

A standard evaluation method has been proposed to assess the matching performance, which is presented as a curve of *recall* versus *1-precision* [41]. The curve is generated below a threshold *t,* which determined whether two descriptors are matched. Given two images representing the same scene, the formulas of *recall* and *1-precision* are depicted in Equation (10):

(10) $$\{\begin{array}{c} recall=N_{1}/N_{2}\\ 1-precision=N_{3}/\left(N_{1}+N_{3}\right) \end{array}$$

where *N*_1_ is the number of correctly point pairs; *N*_2_ is the number of corresponding matched point pairs which are determined by overlapping of the points in different images; and *N*_3_ is the number of the falsely point pairs. The higher *recall* and lower *1-precision* means the better the matching performance. For instance, with the changes of threshold *t*, if *recall* is increasing and *1-precision* is equal to 0, it means that the point pairs are all correctly matched without any falsely matched; if *recall* is static and *1-precision* is increasing, it means that the number of falsely point pairs is increasing, while the correctly point pairs remain unchanged.

In this paper, the PC implementation is used to compare with FPGA. In the PC implementation, the FAST and BRIEF algorithm optimized by OpenCV (2.4.9 version) are calculated in Microsoft Visual Studio 2015 (MVS2015), in which the C++ programming language is adopted. The number of detected points in each image is also defined as approximate 100 and there are 100 point pairs output by the PC.

**Figure 12:**
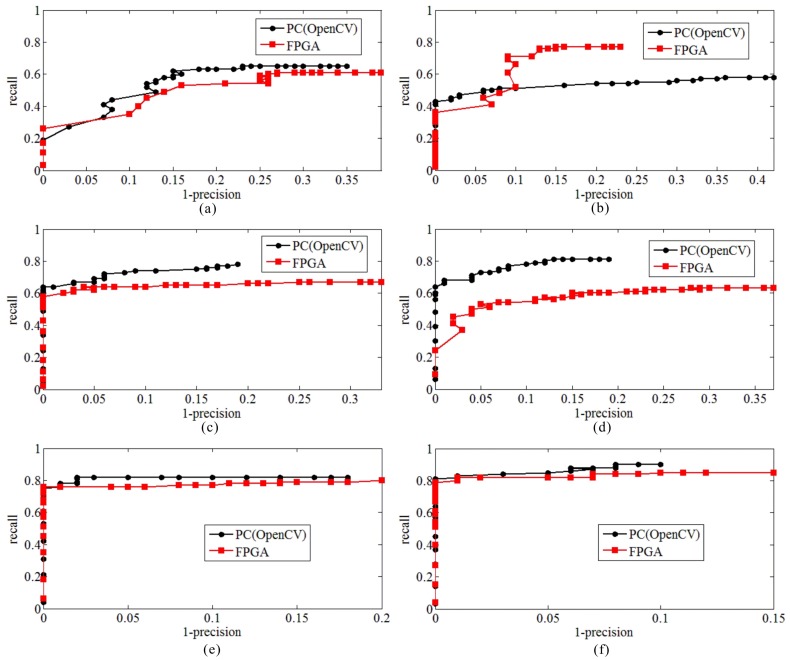
*1-precision* versus *recall* of six image pairs, (**a**) bare soil; (**b**) trees; (**c**) expressways; (**d**) rural roads; (**e**) bungalows; (**f**) high-rise buildings.

The curves of *1-precision versus recall* of the FPGA and the PC implementations with different textures are presented in Figure 12. As seen from Figure 12, the red curve and black curve represent the FPGA’s and the PC’s, respectively. When *1-precision* = 0, the value of *recall* is equal to “c.” It means there are “100*c” correctly point pairs and “0” falsely point pairs, because the maximum number of point pairs is 100 (namely, *N*_1_ + *N*_3_ = 100). The *1-precision* stops at some value “f,” it means that the number of falsely point pairs is “100*f”.

In the bare soil texture (Figure 12a), the two curves are at the similar changes, while the black curve is slightly higher than the red curve. It means that the performance of the PC’s is slightly better than the FPGA’s. Furthermore, the *recall* of red curve and black curve are about 0.24 and 0.20, respectively, when *1-precision* = 0. The *1-precision* of red curve and black curve stop at 0.39 and 0.35.

In the tree texture (Figure 12b), the values of *recall* of two curves are similar when the *1-precision* is equal to 0. When the *1-precision* is greater than 0, the black curve is stable. The red curve has a drastic change when the *1-precision* is between 0.07 and 0.12. The reason is that the number of correctly point pairs is increasing faster than the number of falsely point pairs with the changes of threshold. The *recall* of red curve and black curve are about 0.4 when *1-precision* = 0. The *1-precision* of red curve and black curve stop at 0.23 and 0.43.

In the expressway texture (Figure 12c), two curves keep similar changes, while the black curve is slightly higher than the red curve. The *recall* of red curve and black curve are about 0.6 when *1-precision* = 0. The *1-precision* of red curve and black curve stop at 0.33 and 0.18.

In the rural road texture (Figure 12d), the black curve is higher than the red one, which means that the performance of the PC’s is better than the FPGA’s. The *recall* of red curve and black curve are about 0.22 and 0.62, respectively, when *1-precision* = 0. The *1-precision* of red curve and black curve stop at 0.18 and 0.37, respectively.

In the bungalow and high-rise building textures (Figure 12e,f), the two curves are almost the same, which means that the performances of the PC’s and the FPGA’s are the same in these two textures. In Figure 12e, the *recall* of red curve and black curve are about 0.78, when *1-precision* = 0. The *1-precision* of red curve and black curve stop at 0.18 and 0.20, respectively; In Figure 12f, the *recall* of red curve and black curve are about 0.80, when *1-precision* = 0. The *1-precision* of red curve and black curve stop at 0.15 and 0.10, respectively.

The experiment results indicate that when the images are covered with artificial textures, the performance of the FPGA’s can reach a similar performance as compared with the PC’s, especially in the bungalow and high-rise building textures. The reason is that the artificial textures cover many of robust points, such as inflection points and corners and so forth, which are easy to detect using the FAST detector. In contrast, when images are covered with natural textures, such as trees and rural roads, the performance of the FPGA is poorer than the artificial textures. Furthermore, in natural textures, the performance of the FPGA’s is little poorer than the PC’s, the reason is that the different thresholds are selected for the same texture in the FPGA and the PC.

### 4.4. Speed Comparison and Resource Usage

Speed, as one of the most important factors, needs to be analyzed in an FPGA implementation. In this sub-section, the speed of the CPU, GPU implementation and previous work are compared. First, in comparison with the CPU implementation, a computer with a Windows 7 (64 bit) operating system is equipped with an Intel(R) Core(TM) i7-4790 CPU @ 3.60 GHz and 8 GB RAM. The FAST and BRIEF algorithm optimized by OpenCV (2.4.9 version) is operated in MVS2015. To keep the experiment similar for comparison, the size of the image pair and the number of matching point pairs are defined similarly in this paper. The comparative results are listed in Table 2. As seen in Table 2, the fps of the CPU implementation is 10, while the fps of the FPGA implementation is up to 310, which is a 31 times speedup when compared with the CPU implementation. Second, in comparison with the GPU implementation [42], an Ubuntu Linux 14.10 and an ArrayFire Development Version are used. In hardware, a 32 GB RAM and an NVIDIA K20 GPU is used. An Oriented FAST and Rotated BRIEF algorithm is used in this system. The results indicate that the fps is 125. The speed of the FPGA implementation is 2.5 times as fast as the GPU implementation.

Compared with previous work, this paper and refs. [23,24,25] adopted the same FAST detector, the differences of each are the FPGA architecture. In Table 2, reference [23] only implemented the corner detection on FPGA, where the FAST detector module and the corner score module is executed in parallel. The parallel mode is similar with this paper. The fps of reference [23] reaches 500 with a clock frequency of 130 MHz. Reference [24] firstly achieved the FAST detector module, then achieved corner score module. A serial execution is adopted for saving hardware resources. Hence, when a clock frequency of 100 MHz and a size of 640 × 480, the fps is 55, which is considered low. Reference [25] also implemented the FAST detector module and the corner score module in a sequence. While the FAST module and the BRIEF module are executed in parallel. In addition, there is no DDR3 write/read module. The fps is 325 with a size of 640 × 480, which is highest in these studies. However, the fps in this paper is 310, which is lower than the fps in reference [23,25]. The lower fps is because (1) only corner detection is achieved, such as reference [23]; (2) the size of image and the FPGA architecture are different, such as reference [25]. The proposed architecture aims to balance the speed and data reuse by follow-up algorithms, such as sub-pixel precision location. While the architecture of reference [25] sacrifices data reuse to improve speed, it results in a highest frame rate. Certainly, the speed found in this paper is acceptable in the most satellite applications.

In considering the FPGA resource use, the FPGA resource use of another three studies are used for comparison. In reference [23], when the FAST detector is implemented, the usage of FFT, LUT and RAM are 40%, 62%, 192 kb. In refs. [24,25], the same platform is selected, while the resource use for the FAST and BRIEF is different, the consumption in reference [25] is higher than reference [24], which results in a higher fps in reference [25]. The usage of FFs and LUTs in this paper are 28% and 39%, respectively, while they are approximately 21% and 19% in reference [25]. The use of BRAMs in this paper is approximately 35 kb, while the highest use is up to 1330 kb in reference [25]. In reference [24], the fps is 55 that results in a less use of LUTs and FFTs. Reference [25] and this paper achieved above 300 fps, while this paper cost more hardware resources. The reason is that this paper adds the sub-image write into/read from DDR3 module in FPGA architecture. The higher fps is achieved, the more parallel modules are adopted. The more parallel modules will cost higher hardware resources. Hence, FPGA performance is a set of tradeoffs between hardware resources usage and speed.

## 5. Discussion

In this paper, a new FPGA architecture is proposed for corner detection and matching. A complete evaluation, which considered the different land textures, the accuracy of matching and the speed and resource usage, was initially presented for the FPGA implementation. The experiment results found that the FPGA implementation of the FAST and BRIEF algorithm can reach similar performance when compared with a PC implementation, especially when the image pairs are of buildings. In contrast, when image pairs are of natural textures, a relatively poor performance is presented in the FPGA implementation. This poor performance is due to the images with natural textures having a lack of robust corners and the gray value of the natural textures being similar. The similar gray value will lead to higher mismatching.

In the detection phase, a given threshold *t* was presented in Equation (1) to directly determine the number of detected corners. If the image had natural textures, the threshold *t* was defined as a smaller value to ensure enough corners were detected. While images with artificial textures, the threshold *t* was defined as a larger value to ensure the maximum number of robust corners was detected. Hence, the threshold t was determined based on the texture of the image before processing by the FPGA platform.

In the matching phase, using the PC was the most time-consuming because the codes are executed serially. The issue of time consumption is completely solved using the FPGA. For instance, if there are 100 Hamming distances that need to be compared, a comparison module is executed 100 times on the PC, while in FPGA, 100 comparison modules are executed in parallel. Because of the characteristics of FPGA, such as task parallel processing and pipeline processing, satellite image on-board processing may be possible.

Finally, the FPGA chip (Xilinx XC72K325T FPGA) was selected for this paper because this study is at the stage of a laboratory prototype. The selected FPGA chip may not be suitable for a space environment due to radiation. For an actual space application, the selected FPGA chip must be replaced with a space compatible FPGA chip, such as the Xilinx Virtex FPGA or Actel FPGA.

## 6. Conclusions

A new FPGA hardware architecture for the FAST and BRIEF algorithm is proposed in this paper. In this architecture, the image sequences are sent into the DDR3 for storage and are sent to a detection module for corner detection. With the detected location of the corner, a sub-image centered on the corner is sent into a matching module from the DDR3. During matching, a Hamming distance of two candidate descriptors is calculated and a pair of points is determined by finding a minimum Hamming distance from the candidates.

The high-resolution satellite images located in the Mentougou district, Beijing are selected as the experimental area. Six pairs of images in the experimental area with different textures are used to evaluate the performance FPGA-based behaviors. It can be found from the results of the experiment as follows:

(1) If an image is covered with artificial textures, more robust corners are detected. The value of recall is approximately 0.8 that means the rate of the correct matching of FPGA implementation is same as PC implementation.
(2) The speed of the FPGA implementation is able to reach 310 fps, which is 31 and 2.5 times faster than those of the CPU and of GPU implementation, respectively.
(3) The consumption of the selected FPGA resources is less than 40% that is acceptable for the selected FPGA platform.

## Figures and Tables

**Table 1:** Corner matching from two images (threshold = 3).

ID	Second Image	ID	First Image	XOR	Hamming Distance	Result
1	111111	1	110011	001100	2	Matched
		2	110000	001111	4	Unmatched
		3	010101	101010	3	Unmatched
2	000001	1	110011	110010	3	Unmatched
		2	110000	110001	3	Unmatched
		3	010101	010100	2	Matched
3	110100	1	110011	000111	3	Unmatched
		2	110000	000100	1	Matched
		3	010101	100001	2	Unmatched

**Table 2:** Comparison between proposed method and PC/previous work.

Algorithm	Size	*f* (MHz)	N	fps	Platform	FFTs	LUTs	BRAMs (kb)
FAST+BRIEF(CPU)	512 × 512	3.60 GHz	100	10	Win. 7, i7-4790 CPU	/	/	/
Oriented FAST and Rotated BRIEF (GPU) [42]	512 × 512	706	/	125	Linux, NVIDIA K20 GPU	/	/	/
FAST+BRIEF [This paper]	512 × 512	100	100	310	Xilinx, K7 XC72K325T	112,166	80,472	35
FAST [23]	512 × 512	130	/	500	Xilinx, S3 XC3S200-4	1547	2368	192
FAST+BRIEF [24]	640 × 480	100	/	55	Xilinx, Zynq-7000 SoC	3187	4257	576
FAST+BRIEF [25]	640 × 480	100	100	325	Xilinx, Zynq-7000 SoC	17,412	9866	1330
